# Burden of dengue among febrile patients at the time of chikungunya introduction in Piedecuesta, Colombia

**DOI:** 10.1111/tmi.13147

**Published:** 2018-09-19

**Authors:** Mabel Carabali, Jacqueline K. Lim, Diana C. Palencia, Anyela Lozano‐Parra, Rosa Margarita Gelvez, Kang Sung Lee, Janeth P. Florez, Victor Mauricio Herrera, Jay S. Kaufman, Elsa M. Rojas, Luis Angel Villar

**Affiliations:** ^1^ Global Dengue and Aedes‐transmitted Diseases Consortium International Vaccine Institute Seoul Korea; ^2^ McGill University Montreal QC Canada; ^3^ Universidad Industrial de Santander Bucaramanga Colombia

**Keywords:** dengue, chikungunya, Colombia, disease incidence, fever surveillance, underreporting, clinical characterisation, dengue, chikungunya, Colombie, incidence de la maladie, surveillance de la fièvre, sous‐déclaration, caractérisation clinique

## Abstract

**Objective:**

To estimate the age‐specific incidence of symptomatic dengue and chikungunya in Colombia.

**Method:**

A passive facility‐based fever surveillance study was conducted among individuals with undifferentiated fever. Confirmatory diagnostics included serological and molecular tests in paired samples, and surveillance's underreporting was assessed using capture–recapture methods.

**Results:**

Of 839 febrile participants 686 completed the study. There were 33.2% (295/839) dengue infections (51% primary infections), and 35.9% (191/532) of negative dengue cases there were chikungunya cases. On average, dengue cases were younger (median = 18 years) than chikungunya cases (median = 25 years). Thrombocytopaenia and abdominal pain were the main dengue predictors, while presence of rash was the main predictor for chikungunya diagnosis. Underreporting of dengue was 31%; the estimated expansion factors indicate an underreporting rate of dengue cases of threefold for all cases and of almost sixfold for inpatients.

**Conclusions:**

These findings highlight the ongoing coexistence of both arboviruses, a distinct clinical profile of each condition in the study area that could be used by clinicians to generate a differential diagnosis, and the presence of underreporting, mostly among hospitalised cases.

## Abstract (fr)

**Objectif:**

Estimer l'incidence selon l’âge de la dengue et du chikungunya symptomatiques en Colombie.

**Méthode:**

Une étude de surveillance passive de la fièvre dans les établissements a été menée auprès d'individus présentant une fièvre indifférenciée. Les diagnostics de confirmation comprenaient des tests sérologiques et moléculaires sur des échantillons appariés et la sous‐déclaration dans la surveillance a été évaluée à l'aide de méthodes de capture‐recapture.

**Résultats:**

Sur 839 participants fébriles, 686 ont terminé l’étude. Il y avait 33,2% (295/839) d'infections à la dengue (51% d'infections primaires) et 35,9% (191/532) des cas négatifs pour la dengue étaient des cas de chikungunya. En moyenne, les cas de dengue étaient plus jeunes (médiane = 18 ans) que les cas de chikungunya (médiane = 25 ans). La thrombocytopénie et les douleurs abdominales étaient les principaux facteurs prédictifs de la dengue, tandis que la présence d’éruptions cutanées était le principal facteur prédictif du diagnostic du chikungunya. La sous‐déclaration de la dengue était de 31%; les facteurs d'expansion estimés indiquent un taux de sous‐déclaration des cas de dengue de 3 fois pour tous les cas et de presque 6 fois pour les patients hospitalisés.

**Conclusions:**

Ces résultats mettent en évidence la coexistence continue des deux arbovirus, un profil clinique distinct de chaque condition dans la zone d’étude qui pourrait être utilisé par les cliniciens pour générer un diagnostic différentiel et la présence de sous‐déclaration, principalement parmi les cas hospitalisés.

## Introduction

Dengue (DENV) and Chikungunya (CHIKV) are transmitted to humans by *Aedes* mosquitoes 1, 2. The clinical presentation for both diseases ranges from self‐limiting mild febrile illness to severe forms that in the case of dengue include haemorrhage, shock and death; while for CHIKV a clinical key feature is arthralgia and eventually long‐term arthritis 1, 2, 3, 4. Although a dengue vaccine has been licensed recently in some Latin American countries 5, its use is limited given several considerations of the safety, age of administration, and seroprevalence requirements 5. There is no current specific treatment for either condition and clinical management is substantially symptomatic 1, 2, 3, 4, 6. Despite the fact that the global burden of arboviruses has increased recently 2, 4, 6, dengue incidence remains higher and is still considered a major cause of morbidity and mortality in tropical and subtropical countries 2, 4, 7.

In Colombia, there has been a significant increase in the number of dengue cases during the last 10 years, with epidemic waves occurring every 3–4 years and incidence rates as high as 220 per 100 000 people 8, 9. Dengue epidemiology in Colombia is characterised by the circulation of all four serotypes, and in contrast with Asian countries, the disease occurs in people of all ages 8, 9, 10, 11. Santander, a northeastern province of Colombia, is an endemic area for dengue. Between 2003–2014 the reported annual incidence of DENV ranged from 89 to 1462 per 100 000 people in its metropolitan area, with years 2010 and 2013 considered as epidemic 9, 10. In 2014, CHIKV was introduced in the country and around 25% of 96 687 CHIKV cases notified by the end of 2014 in the country, were from this northeastern region 12, 13.

Outpatient dengue cases account for the greatest burden of disease worldwide; both epidemiologically and economically 1, 2, 7, 9, however there continues to be a lack of data on characteristics of non‐hospitalised patients or about patients receiving care at primary health care facilities 9, 11. To contribute to the evidence of the burden of dengue infection, we estimated the age‐specific incidence of symptomatic dengue cases, characterised its clinical profile, estimated underreporting level, and calculated expansion factors for inpatients and outpatients (IPD/OPD) between 1–55 years of age in Piedecuesta, Colombia. In addition, we contrasted the clinical characteristics and hemogram findings from febrile patients with confirmed diagnosis of dengue and chikungunya in the study setting.

## Methods

### Ethics statement

The study protocol was approved by the Ethical Review Committee of the Universidad Industrial de Santander and by the Institutional Review Board of International Vaccine Institute (IVI). Each participant or legal guardian, in case of minors, provided informed consent.

### Study design and study area

We conducted a passive, facility‐based, fever surveillance study between August 2014 and August 2015 in Piedecuesta, a municipality of the metropolitan area of Bucaramanga, Santander, located at 1000 m.a.s.l, with mean temperature of 24 °C, and relative humidity of 72%. At the time of the study, the catchment population in Piedecuesta was 152 448 in the urban area (306 inhabitants/km^2^) 14. Health insurance coverage is distributed in two main schemes: a government‐subsidised scheme accounting for 55.4% and a contributory scheme comprising 44.4% (for employees or people with capacity to contribute for their health insurance), with just 0.2% of the population without any coverage 14. The study was conducted at the two institutions in which health care attention is concentrated: Piedecuesta's Local Hospital and the Clinic Piedecuesta.

### Eligibility

Residents of Piedecuesta (for at least 6 months) aged 1–55 years who sought care at the study facilities due to undifferentiated fever (temperature ≥37.5 °C by thermometer) of <7 days of duration were eligible. Individuals older than 56 years were excluded due to the low incidence in that age group, and infants under 1 year to the potential presence of maternal antibodies. Patients who reported to have participated of any dengue vaccine trial were also excluded.

### Sampling, enrolment, data collection, and follow‐up

To capture the epidemiology of incident dengue infection in the study area, we intended to enrol every eligible participant presented at the study facilities, to conform our sample. After obtaining informed consent, patients underwent a clinical evaluation conducted by trained study physicians who registered socio‐demographic and clinical characteristics in a standardised case report form used in other DVI studies 15. Individuals were asked to return to the centre for a follow‐up visit 10–14 days after the initial evaluation. A household visit, no later than 21 days from enrolment, was arranged with those participants who did not attend the initially scheduled second evaluation. Re‐enrolment was allowed regardless the laboratory dengue results if the interval between onset of fevers was at least 28 days (Appendix S1).

### Sample collection and laboratory testing

Trained phlebotomists obtained, labelled and stored paired (acute and convalescent/follow‐up) samples for each participant. Samples were centrifuged, separated into cryotubes, and stored at −70 °C for further testing. All acute samples were tested using Dengue Duo^®^ (Standard Diagnostics Inc., Korea), a rapid diagnostic test (RDT) for NS1 antigen, and for dengue immunoglobulin M and G antibodies (IgM/IgG), with a reported sensitivity of 80.7% (95%CI = 75.1–85.7), specificity of 89.1% (95%CI = 81–94.7), and positive predictive value = 94.6% (95%CI = 90.3–97.4) 16. Results from rapid tests were offered to the participants during their first visit. Acute and convalescent samples underwent serologic enzyme‐linked immunosorbent assay (ELISA) testing, specifically Dengue IgM/IgG Capture ELISA PanBio^®^. All serologic‐positive samples were tested with real time reverse transcription‐polymerase chain reaction (*r*RT‐PCR) for confirmation and serotype identification (Appendix S1). Negative and indeterminate results by *r*RT‐PCR were processed by conventional PCR 17, 18. Dengue negative or inconclusive samples (*n* = 532), including a random subsample of dengue positive samples (*n* = 52), underwent IgM/IgG ELISA NovaLisa^®^ and PCR testing for CHIKV 19, 20. Dengue status was assessed using WHO guidelines including confirmatory test results 21, 22, 23.

### Statistical analysis

Summary statistics are presented as medians and interquartile range (IQR), frequencies or proportions. For continuous variables such as age in years, leukocytes and platelets x 10^3^ cells/μl, we performed non‐parametric tests. Multinomial logistic regressions were conducted to estimate the relative risk ratios (RRR) and their corresponding 95% confidence intervals (95% CI) for DENV and CHIKV compared to other cases of undifferentiated fever of unknown etiology. Multivariable binomial regressions (log link) were fitted to estimate risk ratios (RR) for DENV compared to CHIKV, and for severe dengue (as compared to non‐severe dengue). Two distinct models with and without RDTs were fitted, adjusting for socio‐demographic and clinical covariates selected using a stepwise approach based on the Bayesian Information Criterion (BIC). Censoring occurred if the patient was lost‐to‐follow‐up or at the end of the study. Stabilised Inverse Probability Censoring Weighting (IPCW) was used to avoid the potential for selection bias due to loss to follow‐up 24. Overall missing data (leukocytes and platelets [15.4%], presence of comorbidities [6.6%], myalgia [0.8%], arthralgia [0.9%] and abdominal pain [0.6%]), were considered to be ‘completely at random’ (Appendix S1). However, we conducted multiple imputation by chained equations (MICE) to create 20 complete datasets using linear and logistic regression for continuous and binary variables, respectively 25. Although the analysis with complete cases was consistent with that obtained by IPCW adjustment and MICE, here we present the estimates based on multiple imputation. A sensitivity analysis for age distribution was conducted using cubic splines to estimate predicted probabilities for each outcome (Appendix S2). Overall and age‐specific incidence rates per 100‐person years were calculated by using the total and the age‐specific denominators and the number of confirmed dengue cases in eligible individuals as the numerator.

The underreporting level was assessed using capture‐recapture methods 26, comparing laboratory‐confirmed cases in our study (capture) to all notified cases reported during the study period to SIVIGILA, the Colombian national surveillance system (recapture), and estimating age‐specific expansion factors (EF) for both, inpatients and outpatients. The EF is the ratio of the adjusted number of cases obtained from the capture–recapture method, over the reported number of cases to the surveillance system at a given specific period of time. An EF = 1 indicates absence of underreporting and an EF >1 indicates underreporting in the surveillance system 26, 27 (Appendix S3). Data analysis was conducted using Stata 13.1 (StataCorp LP, College Station, TX, USA).

## Results

**Figure 1:**
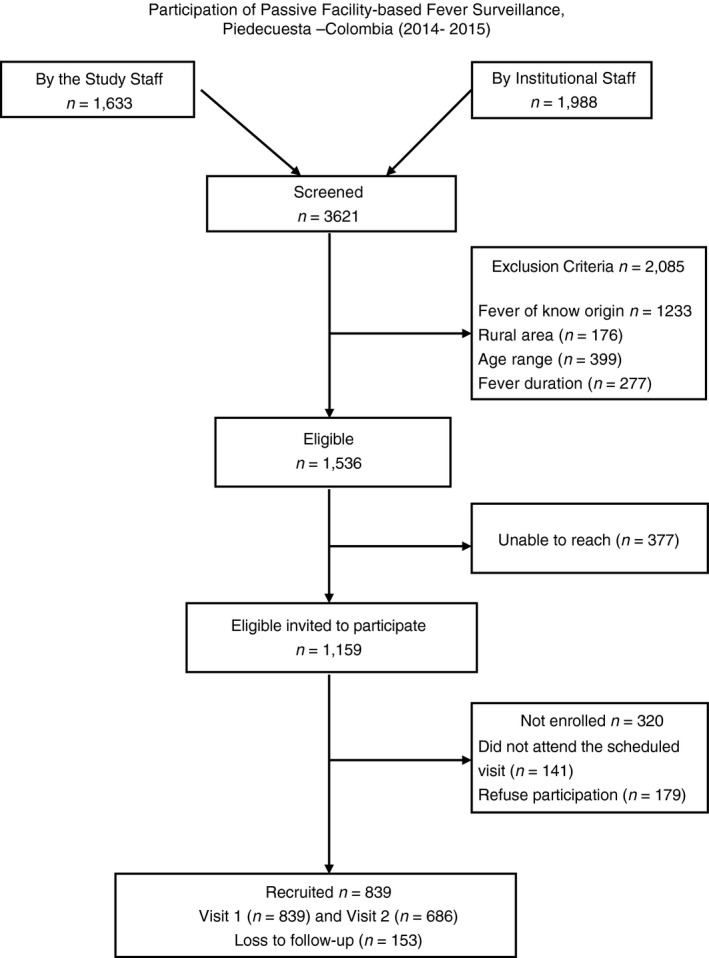
Enrollment and participation flowchart. First day of enrollment August 14, 2015, last day on enrolment July 24th, 2015, and last day of Follow‐up (second visit) August 15 of 2015.

Between August 14, 2014 and July 24, 2015, a total of 3621 people sought health care attention at the study facilities. Of them, 839 participants met the study criteria and were enrolled in our study. There were 153 withdrawals after the first visit and 686 (81.8%) participants completed follow‐up (Figure 1). The median age of the participants was 22 years (IQR 12–33) and of the total, 50.3% (*n* = 422) were men. At enrolment, the median days of fever (DOF) was 4 (IQR 3–4), and 91.4% (*n* = 767) participants were outpatients. Among all participants, 19.6% (*n* = 164) self‐reported previous dengue infection (i.e: self‐reported at least one medical dengue diagnosis in their lifetime) (Table 1).

**Table 1:** General characteristics of participants by dengue and chikungunya status in Piedecuesta – Colombia, (August 2014–August 2015)

	DENV (*n* = 295)	CHIKV (*n* = 191)	Other cause^a^ (*n* = 353)	Total (*n* = 839)
Gender, male	146 (49.5%)	87 (45.5%)	189 (53.5%)	422 (50.3%)
Age, median (IQR)	18.0 (12.0, 29.0)	25.0 (16.0, 38.0)	22.0 (11.0, 32.0)	22 (12, 33)
1–5 years	21 (7.1%)	10 (5.2%)	45 (12.7%)	76 (9.1%)
6–10 years	36 (12.2%)	11 (5.8%)	34 (9.6%)	81 (9.7%)
11–20 years	101 (34.2%)	44 (23.0%)	70 (19.8%)	215 (25.6%)
21–40 years	105 (35.6%)	86 (45.0%)	152 (43.1%)	343 (40.9%)
41–55 years	32 (10.8%)	40 (20.9%)	52 (14.7%)	124 (14.8%)
BMI, median (IQR)	20.7 (17.3, 24.9)	22.9 (19.9, 26.4)	21.6 (17.6, 25.1)	21.4 (16.7, 25.3)
Insurance				
Contributory system	151 (51.2%)	88 (46.1%)	226 (64.0%)	465 (55.4%)
Subsidised system	133 (45.1%)	95 (49.7%)	116 (32.9%)	344 (41.0%)
Out‐of‐pocket	11 (3.7%)	8 (4.2%)	11 (3.1%)	30 (3.6%)
Hospitalisation				
Outpatient (OPD)	218 (73.9%)	188 (98.4%)	337 (95.5%)	743 (88.6%)
Inpatient (IPD)	77 (26.1%)	3 (1.6%)	16 (4.5%)	96 (11.4%)
Days of fever, median (IQR)	8 (6, 10)	7 (5, 9)	5 (4, 7)	7 (5, 9)
<7 days	95 (32.2%)	72 (37.7%)	218 (61.8%)	385 (45.9%)
≥7 days	200 (67.8%)	119 (62.3%)	135 (38.2%)	454 (54.1%)
Previous dengue b	60 (20.3%)	32 (16.8%)	72 (20.4%)	164 (19.6%)
Rash	206 (69.8%)	172 (90.1%)	183 (51.8%)	561 (66.9%)
Myalgia	274 (93.2%)	172 (90.1%)	325 (93.7%)	771 (92.7%)
Arthralgia	239 (81.6%)	180 (94.2%)	303 (87.3%)	722 (86.9%)
Abdominal pain	151 (51.2%)	39 (20.4%)	136 (39.1%)	326 (39.1%)
Nausea/vomiting	223 (75.6%)	133 (69.6%)	233 (66.0%)	589 (70.2%)
Alarm signs c	154 (52.2%)	39 (20.4%)	137 (38.8%)	330 (39.3%)
CNS Alterations d	17 (5.8%)	5 (2.6%)	13 (3.7%)	35 (4.2%)
Positive RDTs e	220 (74.6%)	18 (9.4%)	40 (11.3%)	278 (33.1%)
Comorbidities f	41 (14.9%)	22 (12.3%)	46 (14.0%)	109 (13.9%)
Platelets × 103 cells/μl, median (IQR) g	164 (118, 214)	218 (177, 270)	225 (178, 281)	200 (156, 257)
Thrombocytopaenia h	112 (41.6%)	16 (9.9%)	31 (11.1%)	159 (22.4%)
Leukocytes × 103 cells/μl, median (IQR) g	4.6 (3.6, 6.4)	5.5 (4.3, 6.7)	6.5 (4.8, 9.0)	5.4 (4.0, 7.4)
Leukopenia^i^	105 (39.0%)	35 (21.6%)	39 (14.0%)	179 (25.2%)

a Undifferentiated fever, no dengue and no chikungunya.

b Self‐reported previous dengue infection.

c Any alarm signs described by WHO 2009 guidelines.

d Central Nervous System Alterations such lethargy, seizure.

e Positive dengue RDT (NS1/IgM/IgG).

f Comorbidities including: Diabetes, Hypertension, Cardiovascular diseases, Asthma and allergies.

g Kruskal–Wallis test.

h Thrombocytopaenia: <150 Platelets/μl.

i Leukopaenia: <4.5 × 10^3^ cells/μl.

### Dengue

**Figure 2:**
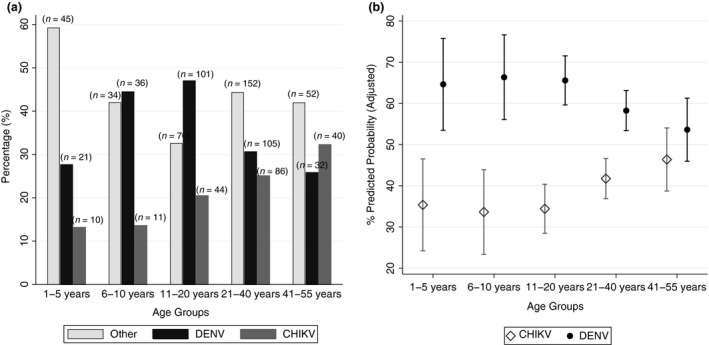
Distribution of DENV and CHIKV cases in Piedecuesta, Colombia (2014–2015). Panel (a) Proportion of laboratory confirmed cases of DENV, CHIKV, or other undifferentiated fever causes by age group. Panel (b) Predicted probabilities and 95%CI of DENV and CHIKV by age. Estimates obtained from the model comparing DENV *vs*. CHIKV (*n* = 486).

There were 295 laboratory‐confirmed DENV infections (35.2%) (Figure 2); the overall median age of dengue cases was 18 years (IQR; 12–29), and 49.5% (*n* = 146) were men. The median platelets (164; IQR = 118–214) and leukocytes (4.6; IQR = 3.6–6.4) level x 10^3^ cells/μl were significantly lower among dengue cases (*P* < 0.001 than among cases of undifferentiated fever (Table 1). A total of 220 samples were positive for any of the dengue NS1/IgM/IgG RDTs, for an overall sensitivity of 74.6% and specificity 89.3% compared to confirmatory analysis.

In the univariate analysis, participants 6–10 and 11–20 years old were relatively more likely to have dengue than participants older than 41 years, OR: 2.3; 95%CI = 1.3–4.2 and OR: 2.6; 95%CI = 1.6–4.1, respectively. However, after adjustment for all other variables throughout all multivariate models, the increased risk of being a dengue case remained statistically significant only among participants 6–10 years old (Table 2), compared to the reference group (41–55 years of age). Likewise, the presence of thrombocytopenia (<150 Platelets/μl) (RRR: 4.1; 95%CI = 2.5–6.8) and abdominal pain (RRR: 1.7; 95%CI = 1.2–2.4) were more likely among patients with a diagnosis of dengue than in those without in all models.

**Table 2:** Multinomial logistic regression models for diagnosis of DENV in Piedecuesta – Colombia, (August 2014–August 2015)

*n* = 839	DENV *vs*. other undifferentiated fever			
Characteristic	Model with RDT		Model without RDT	
Age	RRR	95%CI	RRR	95%CI
1–5 years	0.9	0.4,2.2	0.7	0.3,1.6
6–10 years	1.8	0.8,4.2	2.1	1.0,4.2
11–20 years	1.6	0.8,3.2	1.7	0.9,3.1
21–40 years	1.0	0.5,2.0	1.0	0.5,1.7
41–55 years	Ref	‐	Ref	‐
Gender, male	0.7	0.5,1.1	0.8	0.6,1.1
Insurance				
Contributive	Ref	‐	Ref	‐
Subsidised	1.4	0.9,2.2	1.4	0.9,2.0
Out‐of‐pocket	1.4	0.4,4.2	1.3	0.5,3.4
Leukopaenia	1.4	0.8,2.4	2.3	1.4,3.7
Thrombocytopaenia	2.3	1.3,4.2	4.1	2.5,6.8
Days of fever, >7 days	1.4	0.9,2.1	2.4	1.7,3.5
Positive RDT	14.8	9.2,23.7		
Comorbidities	1.4	0.8,2.6	1.0	0.6,1.7
Abdominal Pain	1.6	1.0,2.5	1.7	1.2,2.4
Rash	1.3	0.8,2.0	1.8	1.3,2.7
Myalgia	0.6	0.3,1.6	0.8	0.4,1.6
Arthralgia	0.9	0.5,1.7	0.8	0.4,1.3

Model 1: Model including RDT; Model 2: Does not include RDT. Thrombocytopenia: <150 Platelets/μl; Leukopenia: <4.5 × 10^3^ cells/μl; Comorbidities including: Diabetes, Hypertension, Cardiovascular diseases, Asthma and allergies.

Of the 295 DENV‐confirmed infections 57.3% (*n* = 169) were primary infections and 16% (*n* = 48/295) were classified as severe cases. All severe cases presented abdominal pain and warning signs; 60.4% (*n* = 29) were males (RR = 1.8; 95%CI = 0.9–3.6) and there was no statistically significant difference between age groups (Appendix S1). Virus identification was possible in 140 samples. DENV1 serotype was the most common at 47.1% (*n* = 66), followed by DENV2 40% (*n* = 56), DENV3 11.4% (*n* = 16) and DENV4 1.4% (*n* = 2).

### Chikungunya

More than a third, 35.9% (191/532) of the dengue‐negative samples tested for CHIKV were laboratory‐confirmed CHIKV infections, of which 54.5% (*n* = 104) were in women. Chikungunya cases were older (median = 25 years; IQR = 16–38) and had a significantly higher proportion of rash and arthralgia than dengue cases (Table 1). Compared to undifferentiated fever, cases of chikungunya were more likely to present rash, have a fever duration longer than 7 days, and belong to a subsidised insurance scheme. The relative risk of chikungunya was significantly lower among participants between 1–5 years of age compared to people between 41–55 years (Table 3).

**Table 3:** Multinomial logistic regression models for diagnosis of CHIKV in Piedecuesta – Colombia, (2014–2015)

*n* = 839	CHIKV *vs*. other undifferentiated fever			
Characteristic	Model with RDT		Model without RDT	
Age	RRR	95%CI	RRR	95%CI
1–5 years	0.2	0.1,0.6	0.2	0.1,0.5
6–10 years	0.5	0.2,1.2	0.4	0.2,1.0
11–20 years	0.5	0.3,1.1	0.5	0.3,1.0
21–40 years	0.6	0.3,1.1	0.6	0.3,1.1
41–55 years	Ref	‐	Ref	‐
Gender, male	0.8	0.5,1.2	0.9	0.6,1.3
Insurance				
Contributive	Ref	‐	Ref	‐
Subsidised	2.2	1.4,3.5	2.2	1.4,3.4
Out‐of‐pocket	1.6	0.5,4.7	1.5	0.5,4.6
Leukopaenia	1.0	0.5,1.8	1.0	0.6,1.8
Thrombocytopaenia	0.9	0.4,1.8	0.8	0.4,1.5
Days of fever, >7 days	2.8	1.8,4.2	2.5	1.6,3.8
Positive RDT	0.5	0.3,1.0		
Comorbidities	0.8	0.4,1.4	0.8	0.4,1.5
Abdominal pain	0.4	0.3,0.7	0.4	0.3,0.6
Rash	10.6	6.0,18.8	9.9	5.7,17.3
Myalgia	0.4	0.2,0.9	0.4	0.2,0.8
Arthralgia	2.1	0.9,4.8	2.3	1.0,5.0

Thrombocytopaenia: <150 Platelets/μl; Leukopaenia: <4.5 × 10^3^ cells/μl; Comorbidities including: Diabetes, Hypertension, Cardiovascular diseases, Asthma, and allergies.

Comparing only DENV to CHIKV, we observed a significantly increased relative risk of DENV among people between 1–20 years of age, compared to people between 41–55 years. Similarly, after adjusting for gender, age and insurance scheme, the presence of Leukopaenia (<4.5 × 10^3^ cells/μl), thrombocytopaenia, and abdominal pain increased the risk of DENV (Appendix S1). In our study population, there was a predicted probability of 66% for DENV among participants between 6–10 years and of 46% for CHIKV among participants between 41 and 55 years (Figure 2). DENV and CHIKV distribution by age as continuous variable using splines followed a similar pattern as that observed using a categorical variable (Appendix S2).

Among people who sought care at study facilities, the highest overall DENV incidence was 47 new cases per 100 persons per year (95%CI = 38.7–57.1), observed in participants 11–20 years old (*n* = 101 cases over 215 person‐years). Among male participants, the highest incidence (44.7 new cases per 100 persons per year; 95%CI = 29.1–68.5) was observed among children 6–10 years old. Chikungunya incidence was slightly greater in women than men, and the highest incidence was observed in women over 40 years old at 41.4 new cases per 100 persons per year (95%CI = 28.8–59.6) (Table 4).

**Table 4:** Incidence (rate per 100‐person years) of dengue and chikungunya by age group and gender in Piedecuesta – Colombia, (August 2014–August 2015)

DENV	Female				Male			
Age group	Person‐time a	*n*	Rate b	95% CI	Person‐time	*n*	Rate	95% CI
1–5 years	32	9	28.1	14.6–54.1	44	12	27.3	15.5–48.0
6–10 years	34	15	44.1	26.6–73.2	47	21	44.7	29.1–68.5
11–20 years	98	52	53.1	40.4–69.6	117	49	41.9	31.7–55.4
21–40 years	183	60	32.8	25.5–42.2	160	45	28.1	21.0–37.7
>41 years	70	13	18.6	10.8–32.0	54	19	35.2	22.4–55.2
Total	417	149	35.7	30.4–42.0	422	146	34.6	29.4–40.7

CHIKV	Person‐time	*n*	Rate	95% CI	Person‐time	*n*	Rate	95% CI
1–5 years	32	3	9.4	3.0–29.1	44	7	15.9	7.6–33.4
6–10 years	34	5	14.7	6.1–35.3	47	6	12.8	5.7–28.4
11–20 years	98	18	18.4	11.6–29.2	117	26	22.2	15.1–32.6
21–40 years	183	49	26.8	20.2–35.4	160	37	23.1	16.8–31.9
>41 years	70	29	41.4	28.8–59.6	54	11	20.4	11.3–36.8
Total	417	104	24.9	20.6–30.2	422	87	20.6	16.7–25.4

a Person‐time = 100‐person/year.

b Rate = number of cases/person‐time.

### Underreporting of dengue

There were 1229 dengue cases (probable and confirmed) notified to SIVIGILA during the study period, however, only 814 were individuals between 1–55 years of age. There were 667 cases captured only by SIVIGILA and 92 cases captured by both, SIVIGILA and our study. There were 48 cases reported to SIVIGILA as dengue cases that were enrolled in our survey but were not confirmed as dengue cases, therefore considered false positive for the capture–recapture underreporting analysis. Of those 48 cases, 16 were confirmed as CHIKV cases, there were no coinfections DENV‐CHIKV. Following the methods described in Appendix S3, we obtained an overall EF = 3.2 for all patients, and an EF = 5.6 for inpatients. The highest EF = 6.7 was observed for individuals older than 40 years old, and the lowest (EF = 2.4) among children between 11–20 years (Table 5).

**Table 5:** Results of capture–recapture analysis for underreporting of dengue cases in Piedecuesta – Colombia (August 2014–August 2015)

Overall	Estimation of dengue cases by				Underreporting
Age	SIVIGILA a	Capture b	Both system	*n* c	EF d
1–5 years	68	21	4	302.6	4.5
6–10 years	91	36	10	308.5	3.4
11–20 years	247	101	41	601.3	2.4
21–40 years	255	105	33	797.1	3.1
>41 years	105	32	4	698.6	6.7
Total	766	295	92	2440.2	3.2

Hospitalised cases	Estimation of dengue cases by				Underreporting
Age	SIVIGILA a	Capture b	Both system	*n* c	EF d
1–5 years	12	4	0	64.0	5.3
6–10 years	24	4	2	40.7	1.7
11–20 years	44	31	5	239.0	5.4
21–40 years	41	29	6	179.0	4.4
>41 years	14	9	0	149.0	10.6
Total	135	77	13	756.7	5.6

a SIVIGILA (Recapture), (Corrected estimates = Cases between 1–55 years, reported between August 2014–August 2015 – False positives (i.e: overall cases reported to SIVIGILA as dengue but that were not dengue confirmed cases in the fever surveillance study).

b Laboratory serologic/molecular confirmed dengue cases by the study.

c Estimated number of total cases, using the formulae for capture –recapture (*n* = [(capture+1)(recapture+1)/(both+1)]‐1].

d Expansion Factor (EF = *n*/SIVIGILA).

## Discussion

This passive facility‐based fever surveillance conducted in Piedecuesta – Colombia allowed identification of incident arboviral diseases in more than half of the participants (*n* = 486; 58%), of which 60% (*n* = 295) were dengue cases. Although the distribution of these diseases could vary according to the setting and the time when the study was conducted 3, 6, 28, this finding aligns with the documented predominant presence of DENV in the study area, and the introduction of CHIKV around the study period 4, 10, 12.

Dengue and chikungunya were identified in individuals of all age groups; however, age‐specific incidence varied within condition and a well‐differentiated age pattern was documented for each disease. Overall, dengue patients were younger than chikungunya patients; therefore, the predicted probability of getting dengue was significantly higher before 20 years of age, with a predicted probability around 70% for people between 6 and 10 years of age. On the other hand, chikungunya cases were more likely to occur in older participants, mostly among people in the 21–40 year age group. This same pattern of age distribution has been observed in an acute febrile illness survey (AFI) conducted in Puerto Rico 28, and in a cross‐sectional study in Brazil 3. Possible explanations include the consideration that older people were susceptible to the recently introduced chikungunya and that older participants were already sensitised by the endemic presence of dengue 2, 4, 23, 28. Chikungunya has been previously documented in older people and speaking for this pattern are (i) an unclear physiological mechanism and (ii) the likelihood of comorbidities at older ages 28, 29, 30, which in turn could be translated into a more‐than‐average‐ symptomatic condition that resulted into a differential heath seeking behaviour pattern. However, in contrast with the reported literature, we did no observe any association between the presence of comorbidities and our studied conditions.

Overall, dengue was identified in 35% of febrile patients who sought care at our study facilities. As classically described 1, 28, 31, 32, 33, 34, predictors for dengue and severe dengue included leukopaenia, thrombocytopaenia, DOF, and abdominal pain. However, leukopaenia and DOF were statistically significant predictors only in the model without RDT results. This could be explained by the adequate performance of the rapid tests in this setting, and the likelihood of clinicians to rely on such a test when they have access to them 35, 36, 37. Thrombocytopaenia and abdominal pain however, were significant and consistent predictors throughout the entire set of our analysis, indicating that regardless of the presence or performance of RDTs, these factors are essential predictors of dengue identification in the clinical practice 3, 11, 32, 34.

Roughly 43% (*n* = 126) of dengue infections were heterotypic secondary infections and 16.3% were severe cases, without any deaths. The majority of severe cases (71%; 34/48) were people between 11–40 years of age, which in concordance with other studies indicates that people will likely be in contact with dengue early in life and could be associated to the history of dengue endemicity in the study area 9, 11. Being female has been traditionally associated with severe forms and it is thought to be related to a differential rate of exposure and to a differential cultural health‐seeking behaviour 1, 8, 9, 38, 39, where women tend to seek more care than men. In our study, however, the higher proportion of severe cases was among males, as was the case of in Puerto Rico 28, which could be associated to a ‘culturally‐driven’ late seeking behaviour among men. Nonetheless, male gender and rash have been reported as early predictors of dengue in Puerto Rico and Vietnam 22, 28, but overall the gender distribution of dengue cases in the Latin American region is diverse 3, 6, 8, 9, 11, 21, 28.

The presence of all four serotypes in the study area, even at the time of chikungunya introduction, confirms an ongoing dengue presence and its endemicity. Although the frequency distribution of dengue serotypes will in general depend of multiple factors including the viral load, patient's DOF, and the method used for identification 17, 23, 32, 33, the higher frequency of DENV1 observed in our study was also observed in other studies conducted in the Americas around the same period 28, which could be associated with a regional pattern of dengue distribution 3, 6, 11, 28, 33.

Compared to dengue, chikungunya was less commonly identified, which is similar to what was reported in Brazil between 2014–2016, and the differences could be attributed to the time and duration of each study 3. Chikungunya was introduced in the Americas in late 2013, in Colombia in mid 2014, and our study was conducted from mid‐2014 to mid‐2015. This timeline gives a relatively reduced opportunity to capture all incident chikungunya cases. Likewise, in the case of Brazil the majority of confirmed cases were dengue and zika, with only a small proportion of chikungunya cases. In which case it is also possible to consider that dengue's force of infection could influence its predominance in Latin America 40. However, given the nature of these studies, these findings are influenced by individual immunological status and a health seeking behaviour that has not been widely studied in our context 4, 6, 41, 42, and therefore should be interpreted with caution.

Overall, chikungunya cases were older than dengue cases, 54.4% were women over 20 years of age, and its main predictor, when compared to dengue, was the presence of rash. These chikungunya predictors have been also described in other settings in the Americas and on the French island of La Reunion 3, 28, 29, 30. Thrombocytopaenia, a key dengue feature, was considered a significant protector of chikungunya in this study. Arthralgia, on the other hand, a classic chikungunya sign, was only associated in the model without RDT. This should emphasise the importance of symptomatology, required to establish differential diagnosis 1, 3, 28. However, this study was planned to identify dengue burden among febrile participants between 1–55 years of age before the introduction of chikungunya, with more resources, and training for dengue identification than for other conditions, which together with the relatively small sample of CHIKV cases may limit the identification of any effect in this respect. 35, 36.

The estimated expansion factors indicate an underreporting rate of dengue cases of threefold for all cases and of almost sixfold for hospitalised patients. Although underreporting for dengue is not an unknown issue 26, 27, 43, this study provides evidence that despite well‐functioning surveillance systems, the dengue burden has been severely underestimated 4, 9, 43. Our study suggests that underreporting is higher for cases under 5‐years and for people older than 41 years of age. This could be attributed to at least two factors, first the identification of dengue in this population, and second the pattern in health seeking behaviour 26, 27, 43. It is known that even in endemic areas, dengue diagnosis in paediatric populations can be challenging in the absence of confirmatory tests, which will in turn limit the opportunity of its notification 23, 32, 33. On the other hand, health seeking behaviour could be different for adults, who will only seek care if the symptoms do not relapse after several days, limiting the opportunity to capture the entire burden of the disease 6, 11, 27, 28, 32. In our study, we used RDTs as a tool to complement our confirmatory diagnostic scheme, however, it could have been used to enhance the detection and therefore the reporting of cases that could have been missed in the absence of a diagnosis tool 36, 37. An additional explanation to the increased rate of underreporting in adults could be the introduction of chikungunya. The majority of chikungunya cases were older, and it is known that its symptoms, especially arthralgia, could generate more disability. Therefore, more attention could have been paid to this population, consequently interfering with the notification process of dengue cases from this population. Although it was expected to have a lower underreporting rate among inpatients, in our study we observed almost a double rate of underreporting compared to outpatient case. This could be related to the confirmation and notification practices according to the national surveillance system 9, 10, 32. In Colombia, it is mandatory to notify all severe cases (suspected and confirmed) to SIVIGILA 10. And is it thought that hospitalised cases are more likely to be severe and be confirmed, compared to outpatient cases 1, 26, 27. However, the fact that in our study not all inpatients were severe cases (48 severe cases/77 inpatients), and that a set of diagnostic tools were in place to either confirm or discard the cases, might have affected the rate of underreporting by decreasing the amount of suspected cases that were usually reported to the surveillance system, with only 13 cases being present in both, capture and recapture systems.

### Strengths and limitations

Our study results are based on 839 febrile patients who were analysed systematically, using a comprehensive set of diagnosis tests, in paired samples, to increase the sensitivity and specificity of the diagnosis, and include estimated levels of underreporting. As in any follow‐up study we experienced some losses to follow‐up that were considered non‐informative by our analysis. However, to control for the possibility of any resulting selection bias, techniques such as IPCW and multiple imputation were used. The introduction of chikungunya during the study period allowed us to compare and identify the epidemiology of these two arboviruses in the study site. Notwithstanding all efforts, these results could be generalisable to the Northeastern region of Colombia but may have limited external validity to other areas with different characteristics. Our study was conducted among febrile patients who voluntarily sought care, using a (passive) facility‐based fever surveillance method to avoid issues of over reporting due to active surveillance 11; therefore, inferences about asymptomatic or mild cases should be limited. Since chikungunya tends to display more symptoms, it is possible that the relative increase in cases among older adults and participants from the subsidised scheme is due to a differential seeking behaviour. It is also important to note that our study included participants up to 55 years of age, and chikungunya cases have been described mostly among people above that age group 3, 6, 12, 29, 30, 41, so no conclusions can be drawn for people over 56 years of age. A sensitivity analysis using splines for age as continuous variable showed a similar age trend than the presented in the main results, indicating that the risk of residual confounding due to the use of age as categorical variable is negligible.

## Conclusions

Dengue is a well‐studied entity and it is known to be endemic in Colombia and other Latin American countries. However, due to the recent introduction of other *Aedes* transmitted diseases, it was thought that its burden may decrease. This study highlights that despite the important level of underreporting, dengue is still an important cause of fever among all age groups in this setting. Likewise, leukopaenia and thrombocytopaenia, which have been traditionally identified as predictors of dengue, now together with abdominal pain, should be considered in the clinical practice for dengue diagnosis, independently of the presence and performance of RDTs. Moreover, dengue and chikungunya are present in Colombia and this study contributes to the evidence of their simultaneous ongoing transmission.

## Supporting information


**Appendix S1.** Supplementary figures and tables.
**Appendix S2.** Sensitivity analysis Using Splines for age effects.
**Appendix S3.** Capture‐Recapture Methods for underreporting estimation.
