# Supplementary material for: Burden of dengue among febrile patients at the time of chikungunya introduction in Piedecuesta, Colombia
Source: Trop Med Int Health. 2018 Sep 19;23(11):1231–41. doi: 10.1111/tmi.13147 (PMC6334506; doi:10.1111/tmi.13147)
Supplement: Supplementary file 1 — Appendix S1. Supplementary figures and tables. Appendix S2. Sensitivity analysis Using Splines for age effects. Appendix S3. Capture‐Recapture Methods for underreporting estimation. [file TMI-23-1231-s001.pdf]

Supplementary Material
Passive Facility-based fever surveillance for Dengue at the time of Chikungunya introduction in Piedecuesta – Colombia

Appendix 1: Supplementary information

Figure S.1. Passive fever surveillance algorithm in Piedecuesta, Colombia (August 2014-July 2015).

Figure S.2. Specimen Testing Algorithm for Dengue and Definition of laboratory-positive and laboratory-confirmed dengue case.

Figure S.3. Estimates and 95%CI from the multivariate binomial regression for DENV compared to CHIKV (coefficients).

Table S1. Complete and missing information by outcome for variables with missing data.

Table S.2 Multivariate binomial regression comparing DENV vs CHIKV only**.**

Table S.3 Multivariate binomial regression comparing Severe dengue vs dengue.

Appendix. 2. Sensitivity analysis Using Splines for age effects

Appendix. 3. Capture-Recapture Methods for underreporting estimation

Supplementary Material
Passive Facility-based fever surveillance for Dengue at the time of Chikungunya introduction in Piedecuesta – Colombia

Appendix 1. A: Enrollment and Follow-up

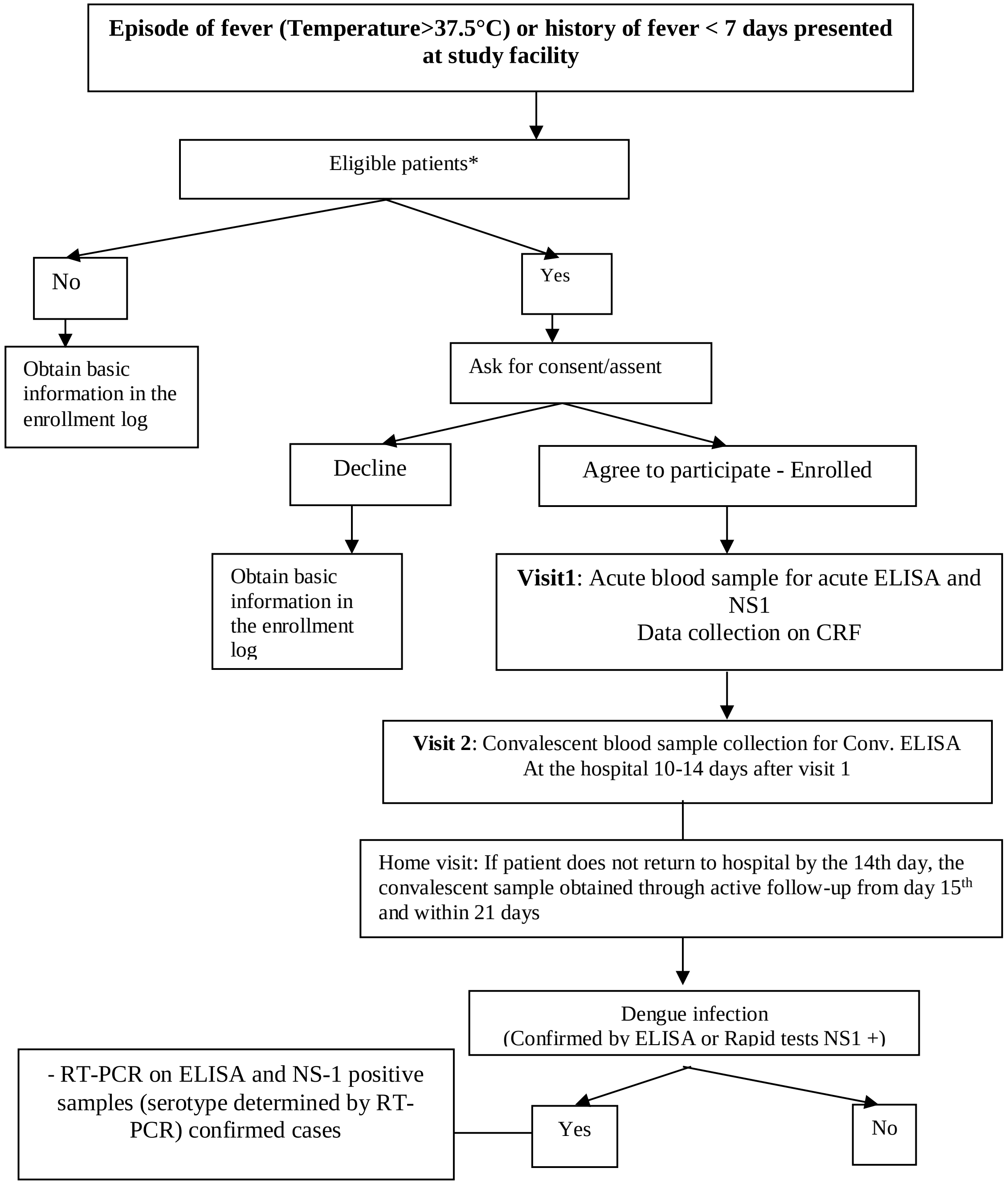

* Eligible participants were those attended by the study staff or by institutional staff if the patient consulted out of the study working ours.

Figure S.1. Passive fever surveillance algorithm in Piedecuesta, Colombia (August 2014- July 2015)

Appendix 1.B: Algorithm Diagnostic

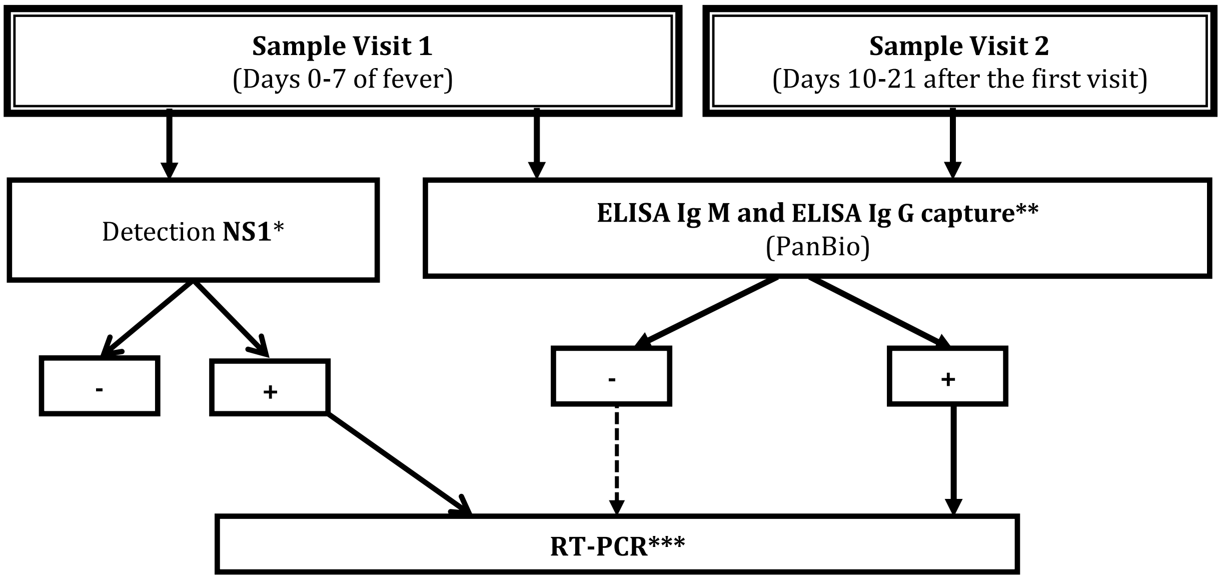

Figure S.2. Specimen Testing Algorithm for Dengue

*NS-1 antigen detection tests would be used to identify dengue cases for immediate follow-up of dengue-confirmed by NS-1/IgM/IgG DengueDuo® (Standard Diagnostics Inc., Korea)
** Dengue IgM/IgG Capture PanBio® All patients underwent Elisa IgM/IgG testing at visit 1 and Visit 2
***IgG positive samples among NS-1 and IgM negative samples with increased antibody titers were tested with RT-PCR. IgG positive samples among NS-1 or IgM positive samples or IgG positive samples only positive during the second visit also underwent molecular testing using RT-PCR.

Definition of laboratory-positive and laboratory-confirmed dengue case

Presence of IgM dengue specific antibody by ELISA in acute (within 7 days of fever) or convalescent (10-21 days after fever onset) serum specimen will indicate dengue-positive case. As we will be using Panbio Dengue IgM capture ELISA test, index value > 1.1, indicated by Panbio unit > 11, would be considered positive; index value 0.9 – 1.1, indicated by Panbio unit 9 – 11, would be considered equivocal (samples require repeat testing); index value < 0.9 indicated by Panbio unit < 9 would be considered negative. In addition, seroconversion of anti-dengue IgM from negative in the acute phase to positive in the convalescent phase and virus detection (RT-PCR) in the acute serum specimen will be considered laboratory-confirmed. For detection of secondary dengue infections among subjects in the surveillance, Panbio IgG Capture ELISA will be used. The index value > 2.2, shown by Panbio unit > 22, would indicate detectable elevated IgG antibodies, identifying that there had been current or recent exposure with dengue virus secondary infections. The index value 1.8 – 2.2, indicated by Panbio unit 18 – 22, would be considered equivocal (require repeated samples testing or further sera collection) and the index value < 1.8 shown by Panbio unit < 18 would indicate absence of detectable elevated IgG antibody levels as evidence of no secondary dengue infection.

Appendix 1.C: Distribution of overall missing data

Table S1. Complete and missing information by outcome for variables with missing data

Variables with missing data	Total (n=839)	DENV (n=295)	CHIKV (n=191)	Other cause (n=353)
**Comorbidities**				
Complete	784	276	179	329
Missing	55	19	12	24
**Myalgia**				
Complete	832	294	191	347
Missing	7	1	0	6
**Arthralgia**				
Complete	831	293	191	347
Missing	8	2	0	6
**Abdominal Pain**				
Complete	834	295	191	348
Missing	5	0	0	5
**Leukocytes (x 10^3 cells/μL)**				
Complete	710	269	162	279
Missing	129	26	29	74
**Platelets (x 10^3 cells/μL)**				
Complete	710	269	162	279
Missing	129	26	29	74

Appendix 1D: Analysis using the multivariate binomial regressions

Table S.2 Multivariate binomial regression comparing DENV vs CHIKV only

DENV vs CHIKV (n=486)	Model with RDT		Model without RDT	
**Characteristic**	**RR**	**95%CI**	**RR**	**95%CI**
**Age**				
1-5 years	2.8	[0.8,9.7]	**3.2**	**[1.1,9.5]**
6-10 years	3.2	[1.0,10.5]	**5.3**	**[2.0,13.7]**
11-20 years	**3.0**	**[1.2,7.4]**	**3.2**	**[1.5,6.6]**
21-40 years	1.5	[0.7,3.5]	1.6	[0.8,3.1]
> 41 years	Ref	[-]	Ref	[-]
**Sex,** Male	0.8	[0.5,1.5]	0.9	[0.6,1.5]
**Insurance**				
Contributive	Ref	[-]	Ref	[-]
Subsidized	0.6	[0.3,1.1]	**0.6**	**[0.3,0.9]**
Out-of-pocket	1.0	[0.2,4.4]	0.8	[0.2,2.8]
**Leucopenia**	1.5	[0.7,3.3]	**2.3**	**[1.3,4.0]**
**Thrombocytopenia**	**3.2**	**[1.4,7.3]**	**6.1**	**[3.3,11.5]**
**Days of Fever**, >7 days	**0.4**	**[0.2,0.8]**	0.9	[0.6,1.5]
**Positive RDT**	**32.9**	**[16.4,66.0]**		
**Comorbidities**	1.7	[0.7,4.0]	1.0	[0.5,2.1]
**Abdominal Pain**	**3.3**	**[1.8,6.1]**	**4.0**	**[2.4,6.5]**
**Rash**	**0.1**	**[0.0,0.2]**	**0.1**	**[0.1,0.3]**
**Myalgia**	1.1	[0.4,3.1]	1.8	[0.7,4.4]
**Arthralgia**	0.5	[0.2,1.3]	**0.4**	**[0.2,0.9]**

Thrombocytopenia: <150 Platelets//µL; Leucopenia: <4.5 x 103 cells/µL; Comorbidities including: Diabetes, Hypertension, Cardiovascular diseases, Asthma and allergies.

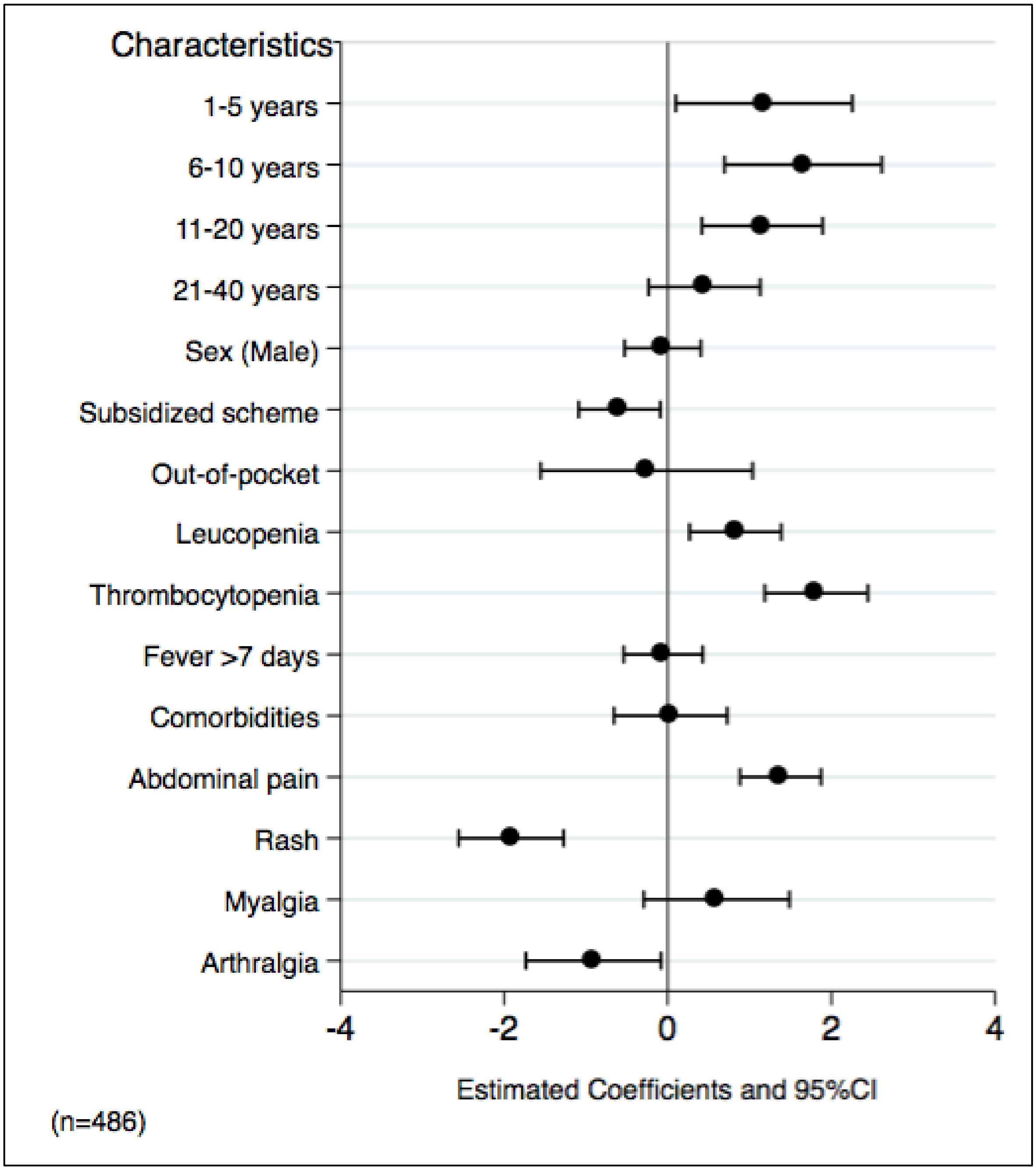

Figure S.3. Estimates and 95%CI from the multivariate binomial regression for DENV compared to CHIKV (coefficients), without adjusting for RDTs. Reference for age is 41-55 years and for Insurance is contributory scheme.

Table S.3 Multivariate binomial regression comparing Severe dengue vs dengue

**Severe dengue vs. Dengue (n=295)**				
**Characteristics**	**RR**	**95% CI**	**Coefficient**	**95% CI**
**Age**				
1-5 years	1.1	[0.2,5.5]	0.10	[-1.6,1.7]
6-10 years	2.0	[0.5,7.8]	0.70	[-0.7,2.1]
11-20 years	1.5	[0.4,4.9]	0.40	[-0.8,1.6]
21-40 years	1.3	[0.4,4.4]	0.30	[-0.9,1.5]
> 41 years	*Ref*	[-]	*Ref*	[-]
**Sex,** Male	1.8	[0.9,3.6]	0.60	[-0.1,1.3]
**Insurance,** Subsidized	0.6	[0.3,1.2]	-0.50	[-1.2,0.2]
**Fever**, >7 days	2.3	[1.0,4.9]	0.8	[0.0,1.6]
**Comorbidities**	0.8	[0.3,2.2]	-0.2	[-1.2,0.8]

Appendix. 2. Sensitivity analysis Using Splines for age effects

The use of splines allows a flexible comparison of the adjusted relationship between age as continuous variable and each outcome.

To estimate the predicted probabilities using splines we proceed with the following steps:

1. Generate the cubic splines using four knots: at age 5, 10, 20, and 40
2. Fit the logistic regression for each outcome, including the splines as covariates
3. Estimate the predicted probabilities using – Adjustrcspline- Stata command
4. Plot the results

Note: since the method for splines do not support weights or MI estimates, this analysis was done with complete cases

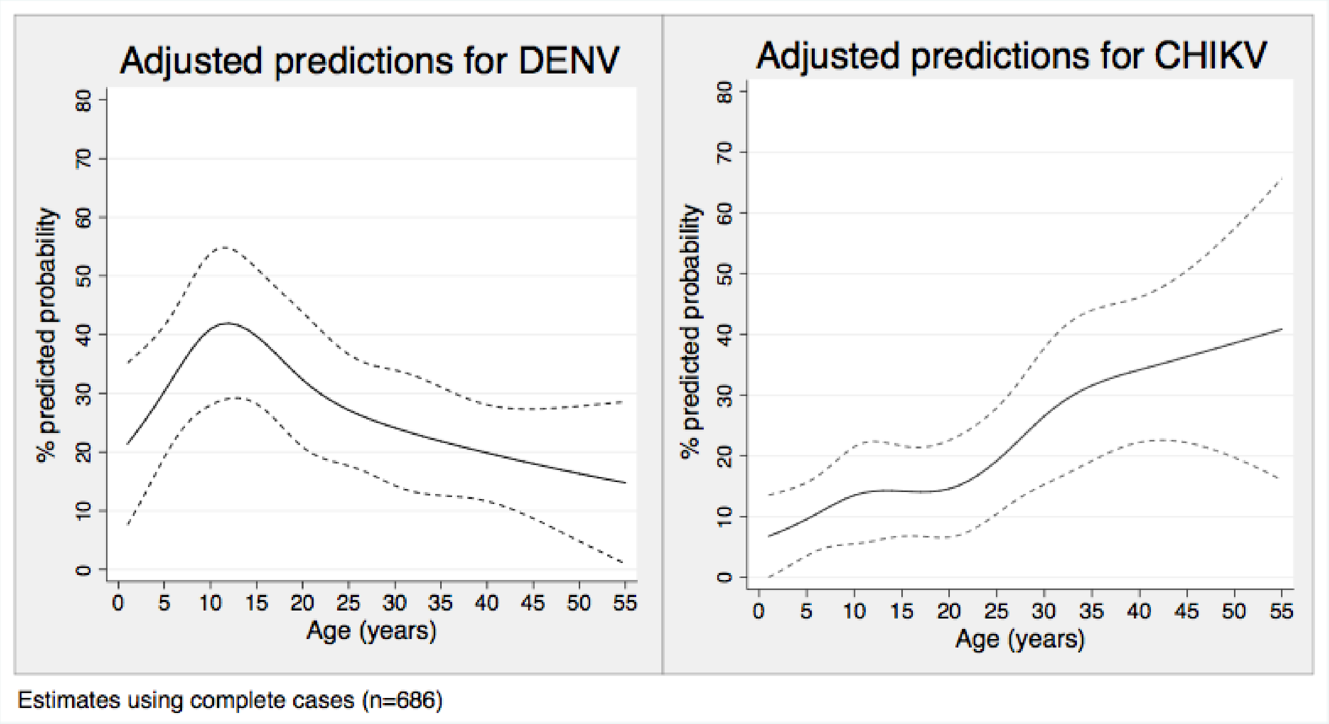

Figure S.3. Predicted probabilities and 95%CI for DENV and CHIKV using splines for age as continuous variable and complete cases. Passive facility-based fever surveillance in Piedecuesta, Colombia (2014-2015)

Appendix. 3. Capture-Recapture Methods for underreporting estimation

Capture- recapture methods involves the evaluation of underreporting by comparing laboratory confirmed cases to surveillance reported data (Vong S et al. 2011). Using the enrollment log for the study, we link the study records to the SIVIGILA system to identify cases captured by both systems (i.e: SIVIGILA and our study), cases enrolled in our study that were not captured by SIVIGILA, and cases enrolled in our study that were capture by SIVIGILA but that were not laboratory confirmed as dengue cases.

To estimate the level of reporting we estimate N, as the estimated total number of dengue cases as follows:

$$N = \left[\frac{(N_A + 1)(N_B + 1)}{X_{AB}} - 1\right]$$

Where N_A is the number of cases by the capture (our study), N_B is the number of cases by the recapture (cases reported to SIVIGILA), and X_{AB}, is the number of cases reported by both, the capture and recapture.
To correct the estimates for False Positives (FP), i.e: reported dengue cases to SIVIGILA, captured by our study that were not confirmed as dengue or chikungunya confirmed cases, we replaced N_B by N_{B^*},
where $N_{B^*} = N_B - FP$

$$N^* = \left[\frac{(N_A + 1)(N_{B^*} + 1)}{X_{AB}} - 1\right]$$

The expansion factor (EF) is the inverse of underreporting rate and it is estimated by the following formulae:

$$EF = {N^*}/{N_{B^*}}$$

Reference:
Vong S, Goyet S, Ly S, Ngan C, Huy R, Duong V, et al. Under-recognition and reporting of dengue in Cambodia: a capture–recapture analysis of the National Dengue Surveillance System. Epidemiology and Infection. 2011;140(3):491-9
